# Beliefs and sharing intentions of human- and AI-generated fake news: Evidence from 27 European countries

**DOI:** 10.1093/pnasnexus/pgag032

**Published:** 2026-02-23

**Authors:** Ádám Stefkovics, Dömötör Gere

**Affiliations:** Szazadveg Foundation, Political Analysis Research Institute, 1037 Budapest, Hungary; ELTE Centre for Social Sciences, CSS-RECENS, 1097 Budapest, Hungary; Institute for Quantitative Social Sciences, Harvard University, Cambridge, MA 02138, USA; Szazadveg Foundation, Political Analysis Research Institute, 1037 Budapest, Hungary

**Keywords:** AI, misinformation, fake news, Russo-Ukrainian war, social sciences, psychological and cognitive sciences

## Abstract

Misinformation remains a major challenge in today’s information environment, and rapid advances in AI-driven content generation risk amplifying this problem. Generative AI represents a double-edged sword: beyond its growing utility for detecting misinformation, it can also facilitate democratic deliberation, counter conspiracy narratives, and promote reliable information, even as the same technologies enable the rapid, large-scale production of persuasive false content. Understanding how people perceive AI-generated misinformation is therefore crucial for designing effective interventions and safeguarding information integrity. To address this, we embedded a preregistered experiment in a large-scale web survey conducted across 27 European countries. Participants were presented with eight short news headlines related to the Russo-Ukrainian war: four AI-generated and four human-generated, evenly split between real and fake news. For each headline, respondents assessed its perceived veracity and their willingness to share it. Our findings show that fake news is consistently viewed as less accurate and less likely to be shared, with systematic differences across countries and individual characteristics such as cognitive reflection, ideology, and trust. While differences between human- and AI-generated content were minimal, the results reveal broader and robust patterns in how people evaluate misinformation across diverse European contexts. These insights highlight the need to strengthen individuals’ cognitive and informational resilience to counter the spread of misleading content in increasingly complex media environments.

Significance Statement Misinformation remains a pressing global issue, and advances in generative AI intensify this threat by enabling the rapid creation of highly convincing false content. We conducted a large-scale experiment across 27 European countries to examine how people perceive human- and AI-generated news about the Russo-Ukrainian war. While fake news is generally viewed as less credible and less shareable, respondents rarely distinguish between human- and AI-generated content. This study provides the first cross-national experimental evidence on these perceptions and explores how cognitive reflection, ideology, and demographics shape responses. The findings highlight the urgent need for interventions that address the unique risks of AI-generated misinformation and strengthen individuals’ capacity to navigate an increasingly complex information environment.

## Introduction

In the contemporary information environment, individuals increasingly rely on the Internet and web-based information, despite its well-documented potential for inaccuracy and bias (1). This reliance occurs against a backdrop of declining public trust in mass media, with the Digital News Report 2025 documenting continued erosion of trust in news across many European countries (2). Misinformation has serious implications for the individual and community as it can influence health behaviors, undermine trust in institutions, and exacerbate social and political polarization (3). Online social networks play an important role in the dissemination of fake news (4). The proliferation of fake news and the opacity of its sources amplify uncertainty regarding social media’s reliability as an information conduit (5). Empirical evidence from cascade-level analyses on Twitter suggests that, within that platform, false political news can diffuse farther, faster, and more broadly than verified information, partly due to its higher perceived novelty (6). Furthermore, online misinformation has become a pervasive constant; only the methods by which actors create and distribute this information are evolving (7).

AI plays a pivotal role in shaping the misinformation landscape (8, 9). Generative AI offers opportunities to strengthen the information environment by supporting democratic deliberation, countering conspiracy narratives, enhancing fact-checking processes, and promoting reliable content (10–12). At the same time, AI can amplify misinformation risks and outpace traditional fact-checking by enabling the rapid, large-scale generation of convincing content (7, 13). Generative technologies such as deepfakes blur boundaries of media authenticity (14), while widespread AI availability lowers barriers for creating and disseminating false narratives (15). Despite these risks, effective detection tools for AI-generated misinformation remain limited (16), and humans often fail to recognize AI-generated texts (7, 17–19).

This study provides the first large-scale, cross-national evidence on how perceived veracity and sharing intentions differ between human- and AI-generated news headlines, across both real and fake content. We ran a preregistered web-based survey experiment in 27 European countries, where 27,000 participants evaluated the perceived veracity and shareability of eight Russo-Ukrainian war headlines. Each respondent saw four AI-generated and four human-generated items, evenly split between real and fake, without knowing the source. We also test whether individual traits (eg cognitive reflections, age, education, and ideology) and regional context moderate main effects.

## Background and hypotheses

Research on individuals’ ability to distinguish real from fake news shows mixed results. While several studies document that people sometimes rate fabricated political headlines as accurate, even when implausible (20, 21), recent meta-analytic evidence indicates that individuals can, on average, differentiate between true and false content better than often assumed (22). This work also suggests that efforts to enhance overall discernment may benefit more from strengthening acceptance of accurate information than from further reducing belief in falsehoods. These challenges are nevertheless amplified in online environments where cues about source credibility are weak, increasing the persuasive potential of misinformation (23).

While extensive research has explored engagement with misinformation more broadly, far fewer studies have examined how people perceive and interact with AI-generated news content, especially when it is misleading or false. AI-generated news often reveals significant skepticism regarding its credibility and authenticity (24, 25). A substantial line of research documents widespread *AI aversion*, showing that people are often uncomfortable with automated systems taking over tasks traditionally performed by humans (26). This reluctance stems from perceptions that AI lacks contextual judgment, empathy, and experiential understanding, leading individuals to trust AI-generated outputs less than those created by humans.

At the same time, various studies highlight that individuals frequently struggle to differentiate between human-written and AI-generated content (7, 17–19), impacting their willingness to engage with or share such information. For instance, Spitale et al. (19) examined users’ ability to determine whether true or false content was produced by humans or AI and found that AI-generated material can be more effective at spreading misinformation than human-written content. They also reported that people rate false Twitter/X posts created by GPT-3 more accurate than human-written posts. In contrast, Bashardoust (13) using a US sample and COVID-19-related fake news, found that AI-generated fake news is perceived as less accurate than human-generated fake news, yet both are shared at similar rates. Other studies labeled true and false news as human- and AI-generated. Both Longoni et al. (27) and Altay and Gilardi (28) using US and UK samples, showed that simply labeling headlines as AI-generated reduces their perceived accuracy and shareability, even when the headlines are true or human-created, reflecting a broader skepticism driven by assumptions of full AI automation. Drawing on these findings, we post the following hypotheses:

*H1a: The perceived veracity will be lower for the fake news than for the real news, regardless of source.*
*H1b: The willingness to share the news item will be lower for the fake news than for the real news, regardless of source.*
*H2a: The perceived veracity of AI-generated news will be lower than that of human-generated news, regardless of whether the news is actually real or fake.*
*H2b: The willingness to share the AI-generated news items will be lower than that of human-generated news, regardless of whether the news is actually real or fake.*

Both the misinformation and AI literature consistently indicate that perceptions of and engagement with fake news and AI-generated content vary across individual characteristics. One line of research shows that socioeconomic factors explain how people engage with AI-generated fake news. For example, older, male, and high-income people tend to better detect fake news (13, 29). Other studies showed that younger people are more receptive to AI-generated content, especially when transparency and readability were prioritized (30). Individual political leanings may also play a role. Some studies show that conservatives are less supportive of AI (31) and that people with moderate or right-leaning political orientations perceive fake news as more credible and are more willing to share it compared to those on the political left (13). Research also shows that cognitive skills are crucial because individuals with higher analytical thinking are better at discerning false information (20). Bashardoust et al. (13) found that those scoring high on the cognitive reflection test (CRT) perceived fake news as less real and were less willing to share it. Similarly, Shin et al. (32) reported that users with strong heuristic–systematic processing abilities and higher perceived diagnosticity are more effective at identifying misinformation than those with weaker cognitive processing skills. Considering the literature, we ask:

*RQ1: Which individual-level characteristics (cognitive reflection, age, education, political ideology) moderate the effects stated in H1 and H2?*

Prior research suggests that susceptibility to and engagement with misinformation vary meaningfully across countries. Variations are expected due to differences in the informational and political landscape (33), media trust (34), digital literacy, platform use (33), value for accuracy (35), uncertainty avoidance, or masculinity (36). Yet, research providing cross-national insights into the interplay between fake news and AI remains scarce. Some studies suggested differences in understanding AI-generated news between Chinese and South Korean (37), and Spanish and Portuguese (38) individuals. In the European context of the Russo-Ukrainian crisis, it is reasonable to assume that societies geographically closer to the conflict may perceive misinformation differently compared to those farther away. Proximity to the conflict can increase emotional involvement, motivation to seek information, and amplify exposure to misinformation. To fill this gap in the literature, we ask the following research question:

*RQ2: Are there any cultural differences in the treatment effects?*

## Results

### Main results

Considering mean differences, participants rated fake news as substantially less credible than real news, with a mean perceived veracity of 2.15 compared to 2.60. Fifty-seven percent of real news headlines were rated as “mostly” or “completely real,” whereas 34% of fake news headlines received these higher credibility ratings. The source had a smaller effect: AI-generated news was perceived slightly more credible than human-written content (2.40 vs. 2.34). Forty-seven percent of AI-generated vs. 44% of human-generated headlines were rated as “mostly” or “completely real.” When combining both factors, real AI-generated news received the highest perceived veracity (2.63), while fake human-written news was rated lowest (2.12).

Smaller differences were observed for sharing intentions. Participants were less willing to share fake news than real news, with a mean difference of 0.18 points on the 4-point scale (1.93 vs. 1.76), and with 34% willing to share real headlines compared to 26% for fake ones. The source of the news did not have an effect on sharing intentions: AI-generated content was rated slightly higher than human-written content (1.86 vs. 1.84), with 31 vs. 29% reporting willingness to share. When combining both factors, real AI-generated news was most likely to be shared (1.94), while fake human-written news was least likely (1.75).

**Fig. 1.**
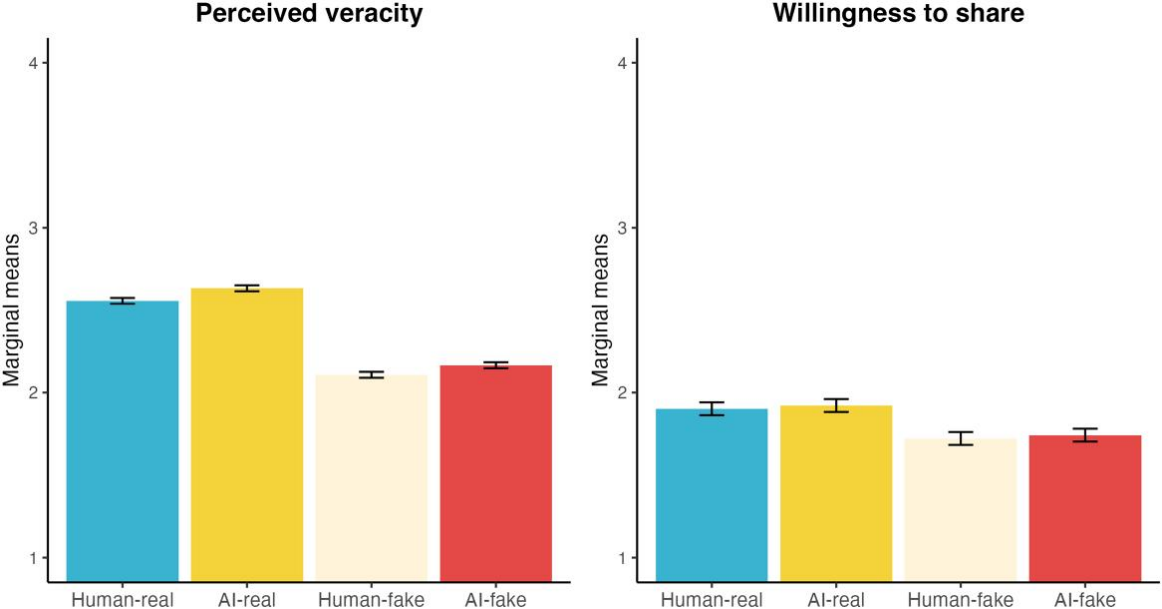
Marginal means of perceived veracity and willingness to share across the four types of news items. Marginal means are derived from multilevel models specified in the Analysis section. Higher values indicate greater perceived veracity and a higher willingness to share. Error bars represent standard errors.

The results of the multilevel models, which account for the clustered structure of the data and control for individual-level characteristics, closely echo these patterns. As shown in Fig. 1 and Table S2, the marginal means of perceived veracity were significantly lower for fake news—especially human-written—compared to real news, with standardized effects of −0.50 (SE=0.01) for *Human-fake* and −0.43 (SE=0.01) for *AI-fake*. In contrast, the effect of source (AI vs. human) was minimal, with a small positive effect for AI-generated content (β=0.08, SE=0.01). A similar but less pronounced pattern emerged for sharing intentions (see Fig. 1 and Table S3): fake news again reduced sharing intent, with standardized effects of −0.20 (SE=0.01) for *Human-fake* and −0.18 (SE=0.01) for *AI-fake*, while the effect of source was nearly negligible (β=0.02, SE=0.01). We fitted the same models among only the respondents who passed the attention check. The results of these models do not alter these findings (see Tables S4 and S5). Altogether, this leads us to the acceptance of H1a and H1b (real vs. fake news), while we found only weak evidence of H2a and H2b (human- vs. AI-generated news).

### Individual moderators

**Fig. 2.**
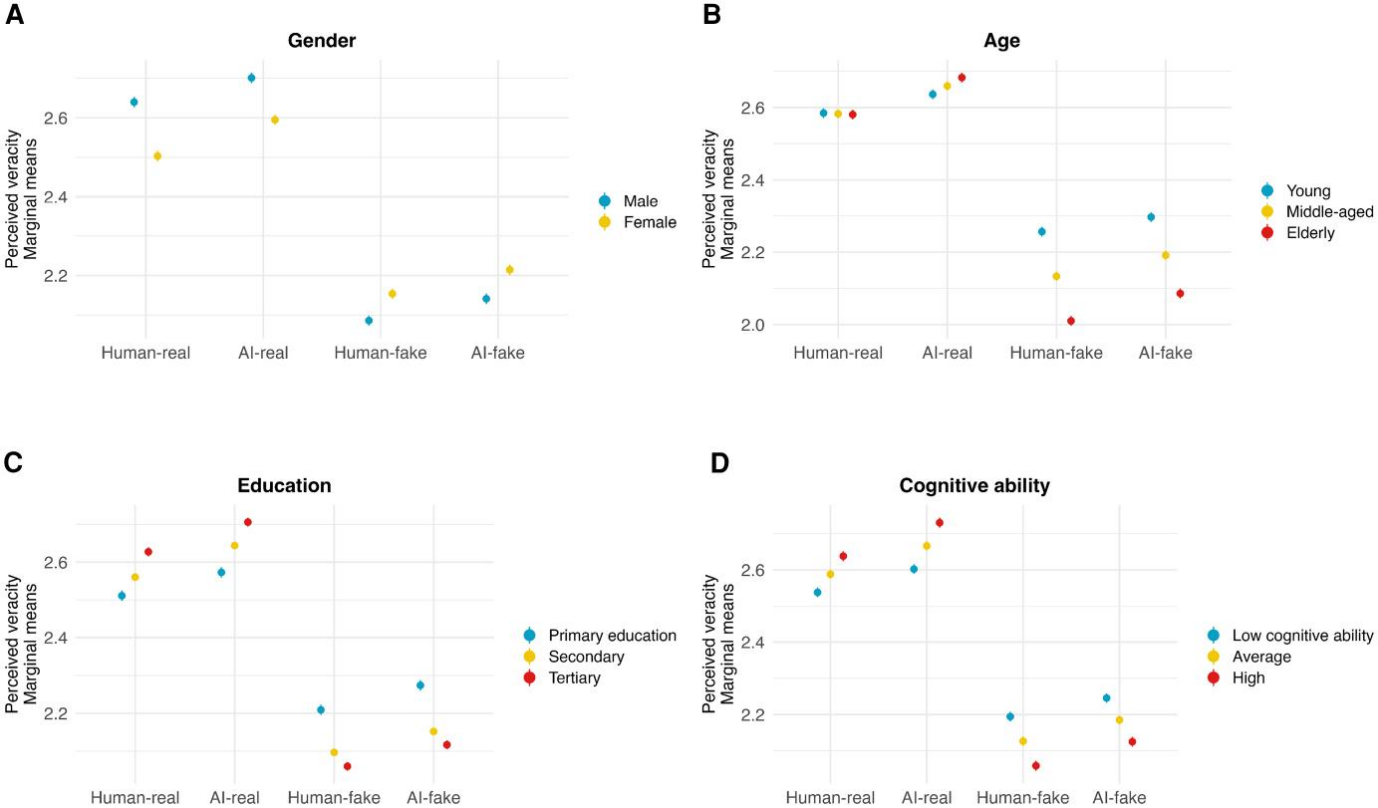
Marginal means of perceived veracity across subgroups. A) Gender differences, B) age differences, C) educational-level differences, and D) cognitive-ability differences. Higher values indicate greater perceived veracity. Error bars represent standard errors. This figure presents a selection of interaction effects with the most substantive differences; see Figs. S1–S7 for all interactions.

Next, we interacted treatment effects with individual characteristics. We plotted the marginal means for each of the interaction effects in Figs. S1–S7. In Fig. 2, we visualized only the interactions that showed substantively large differences. Treatment effects varied by respondents’ gender. Although both men and women rated fake items as less real and were less willing to share them, these effects were notably smaller among female respondents. We also found that perceived veracity scores were more similar for real and fake news items among younger respondents than among older individuals. However, this age-related pattern was not observed for sharing intentions. Highly educated individuals tended to rate real news higher and fake news lower than less educated respondents. Similarly, respondents with lower cognitive reflection showed less differentiation between real and fake items, while those with higher cognitive reflection more clearly distinguished between the two. Treatment effects were consistent across respondents with different political ideologies, financial situations, and settlement sizes. We also highlight that, although perceived veracity varied by individual characteristics, the source of the news—whether human- or AI-generated—had a minimal impact on the responses in all subgroups.

### Country differences

**Fig. 3.**
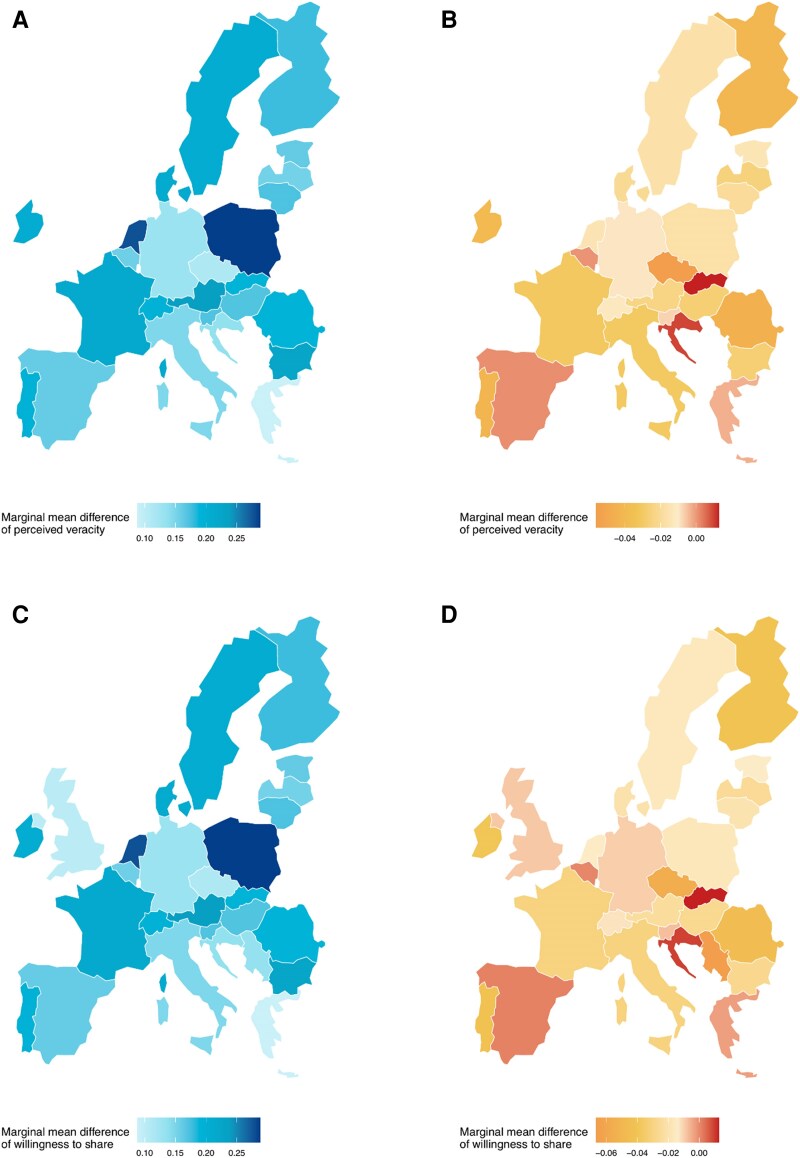
Differences in marginal means of perceived veracity and willingness to share between treatment groups across the 27 countries. A) Perceived veracity comparing real vs. fake news. B) Perceived veracity comparing human- vs. AI-generated news. C) Willingness to share comparing real vs. fake news. D) Willingness to share comparing human- vs. AI-generated news. Higher values indicate that real and human-generated news were associated with higher perceived veracity or willingness to share than fake and AI-generated news.

Lastly, we compare treatment effects across countries. When comparing means, respondents in countries bordering Russia or Ukraine showed remarkably similar patterns of response to those in other countries. For nonborder countries, perceived veracity averaged 2.57 for human-generated real news and 2.64 for AI-generated real news, compared to 2.14 for human-generated fake news and 2.19 for AI-generated fake news. Among respondents in border countries, the pattern was nearly identical: 2.54 for human real, 2.63 for AI real, 2.08 for human fake, and 2.13 for AI fake. These findings confirm that lower perceived veracity and willingness to share fake news relative to real news were consistent across both groups. The results of the multilevel models suggest the same pattern (see Tables S6 and S7). Next, we estimated the same model (without country random effects) in each country. We plotted the differences in marginal means of perceived veracity and willingness to share between real and fake news, and human- and AI-generated news in Fig. 3. The direction of treatment effects is largely consistent across the 27 countries, with substantially higher perceived veracity and sharing intentions for real compared to fake news, although the magnitude of this gap varies across national contexts with no clear regional pattern. By contrast, differences between human- and AI-generated news are minimal and show little systematic variation across countries.

## Discussion

Misinformation remains one of the most pressing challenges in contemporary information ecosystems, and the rapid advances in AI-driven content generation can amplify this threat (7). This study aimed to examine perceptions and sharing regarding human- and AI-generated real and fake news in the Russo-Ukrainian context in a large-scale cross-national survey. Our findings show that people do differentiate between real and fake news, but not between human- and AI-generated news. Regardless of the source, fake news is less likely to be perceived as real and less likely to be shared. This finding is remarkably consistent across the 27 European countries, though some individual characteristics revealed variations.

Our findings regarding fake news’ lower veracity ratings and sharing intention scores resonate well with prior research (39, 40). However, the differences in marginal means are modest (0.45 and 0.18 points on a four-point scale), indicating that while fake news is rated as less credible and less shareable, many respondents still attribute some credibility to fake items, and a substantial share also expresses skepticism toward real news. Importantly, participants were more successful at identifying false news as false than at recognizing true news as true, an asymmetry that aligns closely with recent meta-analytic evidence showing that people tend to under-accept true information more than they over-accept falsehoods (22). This pattern underscores a broader tension between reducing belief in misinformation and avoiding excessive doubt toward accurate content—a dynamic that may be particularly consequential given that audiences encounter far more true than false news in everyday information environments.

Notably, the difference between fake and real news is smaller for sharing intentions than for perceived veracity. The weak correlation between recognizing fake news and the willingness to share it also resonates well with earlier findings (13, 39, 40). However, unlike Pennycook et al. (39) or Bashardoust et al. (13), who reported that many individuals would share fake content even when recognizing it was false, our results show that sharing intention scores were relatively low for both real and fake news. This pattern may signal a broader skepticism toward online news sharing, rather than selective caution toward misinformation alone (22). People in Europe perceive misinformation to be widespread in the context of the Russo-Ukrainian war (34). This may explain why respondents appear reluctant to engage in sharing behavior overall, possibly due to heightened awareness of misinformation risks, normative concerns about spreading inaccurate information, or a lack of trust in online content in general—regardless of its actual veracity. We also note that sharing intentions commonly exhibit floor effects in survey settings and capture a construct that is correlated with, but distinct from, actual sharing behavior, which is shaped by platform dynamics and algorithmic curation.

Consistent with some previous studies (7, 17–19), people did not differentiate much between human- and AI-generated news items. If anything, respondents were slightly more likely to perceive AI-generated content—including fake items—as real and to express willingness to share them. This, to some extent, reinforces the pattern observed by Spitale et al. (19) that AI-generated content can produce more compelling and persuasive disinformation. Moreover, our findings suggest that this applies not only to text-based items but also to stimuli that incorporate AI-generated images, highlighting the broader persuasive potential of multimodal AI-generated misinformation. We note that the source was not disclosed to respondents. It is therefore unclear whether their reactions reflect AI aversion, AI appreciation, or simply an inability to distinguish between human- and AI-generated content. Prior work on labeling content as AI-generated has yielded mixed results: while some studies suggest that respondents may behave more cautiously and trust such items less when they know they are AI-generated (27, 28, 41), others find that labeling media content or messages as AI-generated does not necessarily reduce beliefs or persuasive effects (42, 43), and that, in some cases, corrective messages are more persuasive when labeled as originating from a human expert rather than from AI (44).

Respondents’ gender, age, education level, and cognitive reflection introduced some heterogeneity in the perception of real and fake news items. Consistent with prior research, women, younger and lower educated individuals, and those with worse cognitive reflections were, in some cases, more vulnerable to misinformation. Importantly, though, even subgroups that rated fake items as not real and were the least likely to share them were unable to differentiate between human- and AI-generated content. This suggests that while individual characteristics shape overall susceptibility to misinformation, the challenge of detecting AI-generated news is universal rather than concentrated in specific demographic or cognitive groups.

The findings described above are remarkably consistent across the 27 countries. This is somewhat surprising in light of Hameleers et al.’s (34) results, who found that belief in false information related to the Russo-Ukrainian war is weaker in Northern and Western Europe than in Southern and Eastern Europe. One possible explanation can be that, although individuals encounter misinformation within very different media and political environments, the controlled nature of our stimuli removed contextual signals—such as source credibility, language cues, or ideological alignment—that typically amplify variation across countries. In real-world settings, where these contextual factors are present, differences in how misinformation is perceived are likely to be more pronounced.

Our findings have several implications. First, they reinforce the need for interventions intended to decrease the spread and impact of online misinformation (45, 46). This is because, despite overall skepticism and relatively low sharing intention scores, many respondents still attributed some credibility to fake news (34% of fake news headlines were rated as “mostly” or “completely real”), and even real news items were often met with doubt. Second, the rise of AI-generated misinformation requires novel methods. According to our and others’ (13, 19) results, there is a need for novel cues that users can rely on to distinguish true from fake AI-generated content. Potential additional countermeasures of AI-generated misinformation may include reducing hallucinations, strengthening safety mechanisms, and developing more effective detection algorithms (8). Third, although treatment effects did not vary substantially across countries or individuals in the context of the Russo-Ukrainian war, interventions should still be carefully tailored to specific informational environments and audience subgroups.

Finally, we must acknowledge several limitations of this study. Our analysis focused on a single, highly salient political topic—the Russo-Ukrainian war—where misinformation is both widespread and politically charged. As a result, the findings may be shaped by the specific content, framing, or emotional salience of the news items included. Future research should examine whether similar perceptual patterns emerge across a broader range of topics that vary in politicization and misinformation prevalence, such as health, science, or everyday social issues. Moreover, our experimental design relied on a limited set of stimuli and a controlled survey environment, which may constrain external validity. Some of the null effects—particularly regarding the differences between AI- and human-generated content—may therefore reflect the idiosyncrasies of the selected headlines or model outputs rather than general tendencies. To enhance ecological validity, future studies should incorporate more naturalistic exposure contexts, for instance, by embedding misinformation within simulated or real social media feeds, and by using a more diverse set of AI- and human-produced materials.

## Conclusion

Fake news continues to pose a serious threat to the integrity of public information, and its impact is likely to intensify as generative technologies advance. These tools not only facilitate the detection of misinformation but also enable the rapid, large-scale production of highly convincing fabricated content. Drawing on large-scale, cross-national survey data from 27 European countries, this study demonstrates that GPT-4o models can generate fake news about the Russo-Ukrainian war from a simple prompt, producing items perceived as equally credible—and in some cases even more so—than human-written news. The results underscore how convincingly fake news can mimic real information and highlight the need for interventions that enhance individuals’ resilience to deceptive content in AI-driven media environments.

## Materials and methods

### Data collection

The data come from a large-scale web survey conducted in 27 European countries between 2025 May 5 and June 13. The sample was provided by CINT. Respondents were members of nonprobability-based online panels. The target sample size was 1,000 in each country; the total sample size is 27,227. Sample sizes were determined based on rationales external to this study. We did not use proportional weighting that accounts for differential country population sizes because our analyses focus on individual-level relationships rather than producing population-representative cross-national estimates. With sample sizes of 1,000 respondents per country, the experiment is well-powered to detect even small differences and allow testing interaction effects. Quota sampling was used, with quotas established for gender, age, education, and region (NUTS2 level).

To adjust for nonresponse and the remaining imbalances in demographic representation, we applied post-stratification weights based on age group, gender, education, and region (NUTS2). Population benchmarks were primarily drawn from the European Union Labour Force Survey and the European Social Survey. Due to a small amount of missing data in the weighting variables, weights could only be applied to 26,437 respondents. This constitutes the effective sample size used in our analyses.

The experiment was embedded in a 20-min-long questionnaire. Questions not addressed in this study pertained to attitudes toward national sovereignty. The experiment was placed in the first section of the questionnaire.

### Experimental design

We implemented a fully balanced within-subjects experimental design to examine the effects of source (human- vs. AI-generated) and veracity (real vs. fake) on participants’ responses to news content. The stimulus set consisted of 24 distinct news items, systematically constructed to vary along two orthogonal dimensions: production source (human-written vs. AI-generated) and veracity (real vs. fabricated). This resulted in a 2 (source) × 2 (veracity) factorial structure. Each participant was randomly assigned a subset of eight news items from the full set, resulting in 8,000 evaluations per country. The composition of this subset was fixed such that every respondent evaluated exactly two items from each of the four experimental conditions: (i) human-written real news, (ii) human-written fake news, (iii) AI-generated real news, and (iv) AI-generated fake news. Aside from this constraint, news items were randomly drawn for each participant to ensure item-level randomization and minimize systematic biases. To prevent redundancy and artificial inflation of similarity judgments, we introduced a further constraint: participants could not be exposed to both the human- and AI-generated versions of the same base story. With this, we intended to avoid demand effects and preserve internal validity. We acknowledge, however, that this design, to some extent, limited our opportunities for causal inference because it prevents within-person comparisons of reactions to alternative versions of the same underlying stimulus.

The news items were all about the Russo-Ukrainian war. We chose to keep the topic constant across all items to avoid confounding effects from issue salience, prior knowledge, or topic-specific biases. Given that the design did not allow for systematic variation across topics, thematic consistency was key for internal validity. The Russo-Ukrainian war was selected because it represents a highly salient and information-rich context in which misinformation is widespread and the boundary between authentic and fabricated content is often blurred (47). We first selected eight real and eight fake news items. Real news items were drawn from verified news outlets, while fake items were adapted from known misinformation sources or fabricated based on recurring false narratives. Each item was selected to highlight a different aspect of the conflict, such as military developments, communication on social media or claims of political leaders. Each item was accompanied by a photo from the original news source, or, if unavailable, by a thematically relevant image from another human-generated source.

**Fig. 4.**
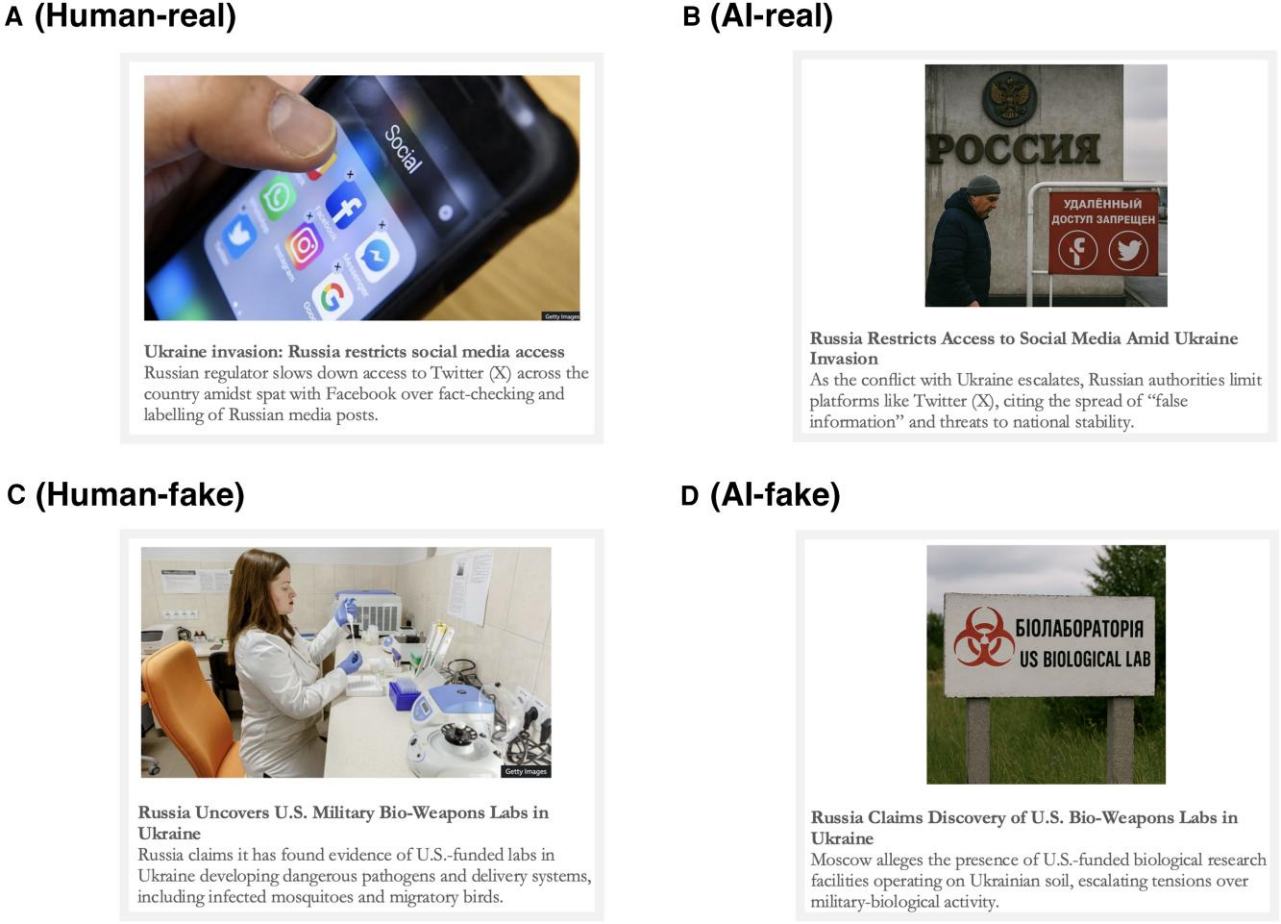
Examples of news item stimuli across the four experimental conditions. A) A human-generated real news item. B) An AI-generated real news item. C) A human-generated fake news item. D) An AI-generated fake news item. See the Supplementary information for the full set of stimuli. Source for the photo in Panel A: https://www.bbc.com/news/technology-60533083. Source for the photo in Panel C: https://www.bbc.com/news/60711705.

In the next step, we generated an “AI version” of these news items. We used OpenAI’s GPT-4o model to generate news content. The model was provided with a prompt in the following format: “Here is a news headline. Use this to generate a short news item with a similar title and some more text that elaborates on the title and gives more detail. Generate a photo as well, which illustrates the news item.” Based on this input, the model produced a headline, a brief accompanying text, and a synthetic image. The news items were originally produced in English and subsequently translated into each country’s language by local experts, following the same procedure used for the main questionnaire. Examples of the stimuli is presented in Fig. 4. All news items are presented in the Supplementary information.

Participants were first instructed that they would be shown eight short news items about the Ukrainian-Russian war. After each news item, two questions were asked of them. We first assessed perceived veracity by asking “To what extent do you believe the news item you just read is real?” with a four-point scale ranging from “Does not seem real at all” to “It seems completely real.” Next, we assessed their willingness to share the news items by asking “How likely is that you would share the news item you have just read on social media?,” with responses ranging from “Definitely would not (1)” to “Definitely would (4).” We employed a “skipping-allowed” design, ie respondents were allowed to skip the questions without answering, but nonresponse options were not displayed visually. As shown in Table S1, skipping was infrequent, below 1% for both DVs and across all experimental groups, thus missingness did not introduce substantive measurement error. After evaluating the eight items, participants received an attention check question (see the full questionnaire in the preregistration) and were debriefed with a message informing them that some of the news items they had read contained false claims and that the experiment also included news generated by AI.

The experiment was preregistered. The preregistration can be viewed under the following link: https://doi.org/10.17605/OSF.IO/XDA3N.

### Other measures

We applied several measures of individual characteristics that may correlate with information processing. The respondents’ gender was measured in a binary way. Age was measured with a continuous variable. Education was measured with “Primary,” “Secondary,” and “Tertiary” categories. We also included a measure of settlement size, with a five-grade scale ranging from “Big city” to “Rural property.” Subjective financial situation was measured with a scale ranging from “We are finding it very difficult to live on our current income (1)” to “We live comfortably on our current income (4).” Political ideological orientation was measured using a five-point scale ranging from “Strongly liberal” to “Strongly conservative.” We also included the CRT (48) to measure the tendency or capacity to pause and critically evaluate a question rather than immediately providing an intuitive or initial answer.

### Analysis

To test the main effects (H1 and H2), we first compare means across the four conditions for the two dependent variables (perceived veracity and sharing intentions). Evaluations are nested within individuals and countries. To obtain a more robust estimate of the treatment effects, we fitted multilevel linear regression models, as specified in the preregistration. The models include random intercepts for both respondents and countries. We include the treatment as fixed effects and other individual-level characteristics for controlling purposes. Specifically, we estimate the following linear multilevel model for news item *i*, respondents *j*, and country *k*:


(1) $$\begin{array}{rl} Y_{ijk} & =\beta_{0,jk}+\beta_{1}{\text{Treatment}}_{\;jk}+\beta_{1}{\text{Gender}}_{\;jk}+\beta_{2}{\text{Age}}_{\;jk}\\ & \;+\beta_{3}{\text{Edu}}_{\;jk}+\beta_{4}\text{Settlement}\;jk+\beta_{5}\text{Financial}\;jk\\ & \;+\beta_{6}{\text{Ideology}}_{\;jk}+\beta_{7}{\text{Cognitive}}_{\;jk}+\varepsilon_{ijk}, \end{array}$$

where $Y_{ijk}$ denotes the evaluation of news item *i* by respondent *j* in country *k*, and $\varepsilon_{ijk}$ is the individual-level residual.

The random intercept is modeled as:


(2) $$\beta_{0,jk}=\gamma_{00}+u_{0j}+v_{0k},$$

where:

$\gamma_{0,0}$ is the overall intercept,

$u_{0j}∼N(0,\sigma_{u}^{2})$ is the random effect for respondent *j*,

$v_{0k}∼N(0,\sigma_{v}^{2})$ is the random effect for country *k*,

$\varepsilon_{ijk}∼N(0,\sigma^{2})$ is the level-1 residual error.

To test whether the treatment effects are moderated by some of the individual characteristics (RQ1), we use the same model specification and add interaction terms between the treatment and relevant individual-level variables.

Lastly, we present marginal means across the four treatment conditions in countries bordering Ukraine or Russia and in nonbordering countries (RQ2). We also estimate each model in each country, excluding the country random effect.

We used Rstudio and the *lme4* (49) and *ggeffectcs* (50) packages for modeling.

## Supplementary Material

pgag032_Supplementary_Data

## Data Availability

The data and code used for the analysis are available on the OSF site https://osf.io/snj29/overview.

## Preprints

This manuscript was posted on a preprint: https://osf.io/preprints/socarxiv/spdb6_v1.
