# Supplementary material for: Beliefs and sharing intentions of human- and AI-generated fake news: Evidence from 27 European countries
Source: PNAS Nexus. 2026 Feb 23;5(3):pgag032. doi: 10.1093/pnasnexus/pgag032 (PMC12964124; doi:10.1093/pnasnexus/pgag032)
Supplement: pgag032_Supplementary_Data [file pgag032_supplementary_data.zip › PNASNEXUS-PNASNEXUS-2025-01175-TRRR-s03.pdf]

Supplementary Information

Beliefs and Sharing Intentions of Human- and AI-Generated Fake News: Evidence from 27 European Countries

Ádám Stefkovics[a, b, c,*] and Dömötör Gere[a]

[a]Századvég Foundation, Budapest, Hungary
[b]ELTE Centre for Social Sciences, Budapest, Hungary
[c]Institute for Quantitative Social Sciences, Harvard University, Cambridge, MA, USA
[*]Corresponding Author: stefkovics.adam@tk.hu, Budapest, Hungary, 1097, Toth Kalman u. 4.

Missingness in the dependent variables

Table S1. Proportion of missingness for the dependent variables across the experimental groups

	**Perceived veracity (%)**	**Willingness to share (%)**
Total sample	0.32	0.62
Human-real	0.31	0.65
AI-real	0.31	0.61
Human-fake	0.31	0.59
AI-fake	0.36	0.64

Main effects

Table S2. Results of the linear multilevel regression predicting perceived veracity

	**b (SE)**	**Std. Beta (SE)**	*p*
Intercept	2.65 (0.02)	0.23 (0.01)	< 0.001
AI-real (ref.: Human-real)	0.08 (0.00)	0.08 (0.01)	< 0.001
Human-fake	-0.45 (0.00)	-0.50 (0.01)	< 0.001
AI-fake	-0.39 (0.00)	-0.43 (0.01)	< 0.001
Gender (ref.: Male)	-0.03 (0.01)	-0.03 (0.01)	< 0.001
Age	-0.26 (0.01)	-0.06 (0.00)	< 0.001
Secondary education (ref.: Low)	-0.03 (0.01)	-0.03 (0.01)	0.004
Tertiary education (ref.: Low)	-0.01 (0.01)	-0.02 (0.01)	0.178
Settlement size	0.03 (0.01)	0.01 (0.00)	0.002
Financial situation	-0.02 (0.01)	-0.01 (0.00)	0.083
Liberal-conservative	0.06 (0.02)	0.01 (0.00)	< 0.001
Cognitive ability	-0.01 (0.01)	-0.00 (0.00)	0.259
**Random Effects**			
σ^2		0.61	
τ_{00} (Respondents)		0.11	
τ_{00} (Country)		0.00	
ICC		0.16	
N (Respondents)		25,605	
N (Country)		27	
N (Evaluations)		203,651	
R^2 (Marg. / Cond.)		0.073 / 0.218	

Table S3. Results of the linear multilevel regression predicting willingness to share

	**b (SE)**	**Std. Beta (SE)**	*p*
Intercept	2.29 (0.03)	0.22 (0.03)	< 0.001
AI-real (ref.: Human-real)	0.02 (0.00)	0.02 (0.00)	< 0.001
Human-fake	-0.18 (0.00)	-0.20 (0.00)	< 0.001
AI-fake	-0.16 (0.00)	-0.18 (0.00)	< 0.001
Gender (ref.: Male)	-0.12 (0.01)	-0.14 (0.01)	< 0.001
Age	-0.78 (0.02)	-0.19 (0.00)	< 0.001
Secondary education (ref.: Low)	-0.10 (0.02)	-0.12 (0.02)	< 0.001
Tertiary education (ref.: Low)	-0.12 (0.02)	-0.13 (0.02)	< 0.001
Settlement size	0.12 (0.01)	0.04 (0.00)	< 0.001
Financial situation	-0.02 (0.01)	-0.01 (0.00)	0.136
Liberal-conservative	0.07 (0.02)	0.02 (0.00)	0.001
Cognitive ability	-0.33 (0.01)	-0.10 (0.00)	< 0.001
**Random Effects**			
σ^2		0.36	
τ_{00} (Respondents)		0.35	
τ_{00} (Country)		0.01	
ICC		0.50	
N (Respondents)		25,605	
N (Country)		27	
N (Evaluations)		203,651	
R^2 (Marg. / Cond.)		0.062 / 0.533	

Table S4. Results of the linear multilevel regression predicting perceived veracity among those who passed the attention check

	**b (SE)**	**Std. Beta (SE)**	p
Intercept	2.67 (0.03)	0.30 (0.02)	< 0.001
AI-real (ref.: Human-real)	0.11 (0.01)	0.12 (0.01)	< 0.001
Human-fake	-0.60 (0.01)	-0.66 (0.01)	< 0.001
AI-fake	-0.49 (0.01)	-0.55 (0.01)	< 0.001
Gender (ref.: Male)	-0.03 (0.01)	-0.03 (0.01)	0.010
Age	-0.22 (0.02)	-0.05 (0.01)	< 0.001
Secondary education (ref.: Low)	-0.02 (0.02)	-0.02 (0.02)	0.223
Tertiary education (ref.: Low)	-0.01 (0.02)	-0.01 (0.02)	0.737
Settlement size	0.02 (0.02)	0.01 (0.01)	0.212
Financial situation	0.01 (0.02)	0.00 (0.01)	0.670
Liberal-conservative	0.05 (0.03)	0.01 (0.01)	0.060
Cognitive ability	0.00 (0.02)	0.00 (0.01)	0.879
**Random Effects**			
σ^2		0.58	
τ_{00} (Respondents)		0.08	
τ_{00} (Country)		0.00	
ICC		0.13	
N (Respondents)		8,161	
N (Country)		27	
Observations		65,090	
R^2 (Marg. / Cond.)		0.125 / 0.236	

Table S5. Results of the linear multilevel regression predicting willingness to share among those who passed the attention check

	**b (SE)**	**Std. Beta (SE)**	p
Intercept	2.13 (0.05)	0.28 (0.04)	< 0.001
AI-real (ref.: Human-real)	0.03 (0.01)	0.03 (0.01)	< 0.001
Human-fake	-0.21 (0.01)	-0.26 (0.01)	< 0.001
AI-fake	-0.18 (0.01)	-0.22 (0.01)	< 0.001
Gender (ref.: Male)	-0.14 (0.01)	-0.16 (0.02)	< 0.001
Age	-0.64 (0.04)	-0.16 (0.01)	< 0.001
Secondary education (ref.: Low)	-0.09 (0.03)	-0.11 (0.04)	0.004
Tertiary education (ref.: Low)	-0.08 (0.03)	-0.10 (0.04)	0.008
Settlement size	0.13 (0.02)	0.05 (0.01)	< 0.001
Financial situation	-0.01 (0.02)	-0.00 (0.01)	0.743
Liberal-conservative	0.08 (0.04)	0.02 (0.01)	0.022
Cognitive ability	-0.30 (0.02)	-0.11 (0.01)	< 0.001
**Random Effects**			
σ^2		0.29	
τ_{00} (Respondents)		0.31	
τ_{00} (Country)		0.01	
ICC		0.52	
N (Respondents)		8,161	
N (Country)		27	
Observations		65,090	
R^2 (Marg. / Cond.)		0.062 / 0.551	

Interactions

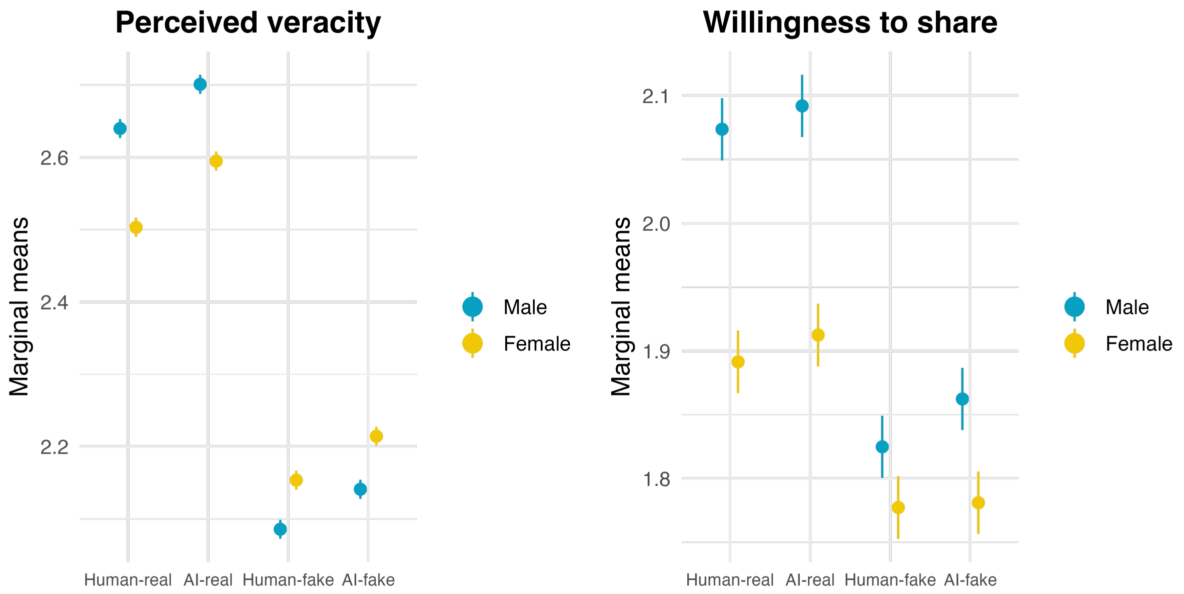

Fig S1. Marginal means of perceived veracity and willingness to share between genders
Note: Higher values indicate greater perceived veracity and a higher willingness to share. Error bars represent standard errors.

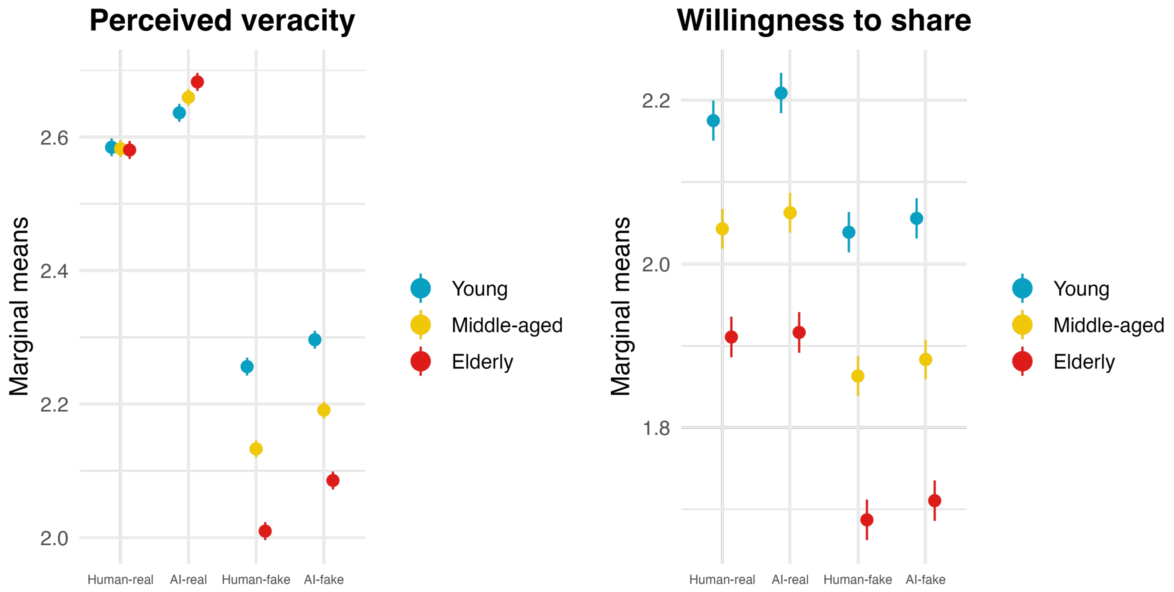

Fig S2. Marginal means of perceived veracity and willingness to share by age-groups
Note: Higher values indicate greater perceived veracity and a higher willingness to share. Error bars represent standard errors. Differences between age groups were assessed using the mean and standard deviation.

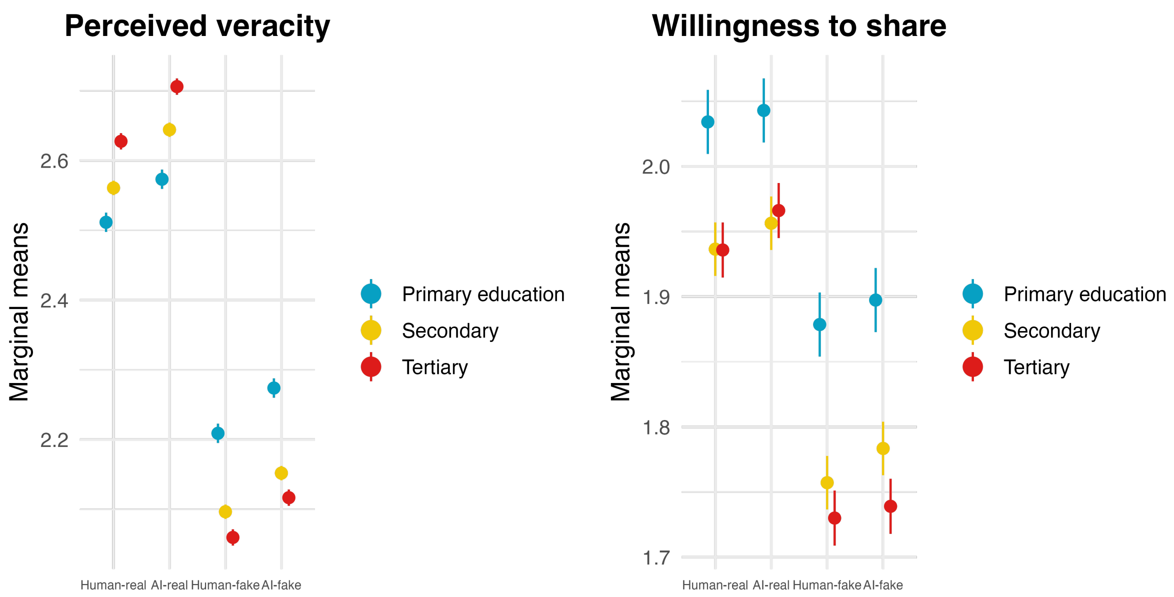

Fig S3. Marginal means of perceived veracity and willingness to share by education
Note: Higher values indicate greater perceived veracity and a higher willingness to share. Error bars represent standard errors.

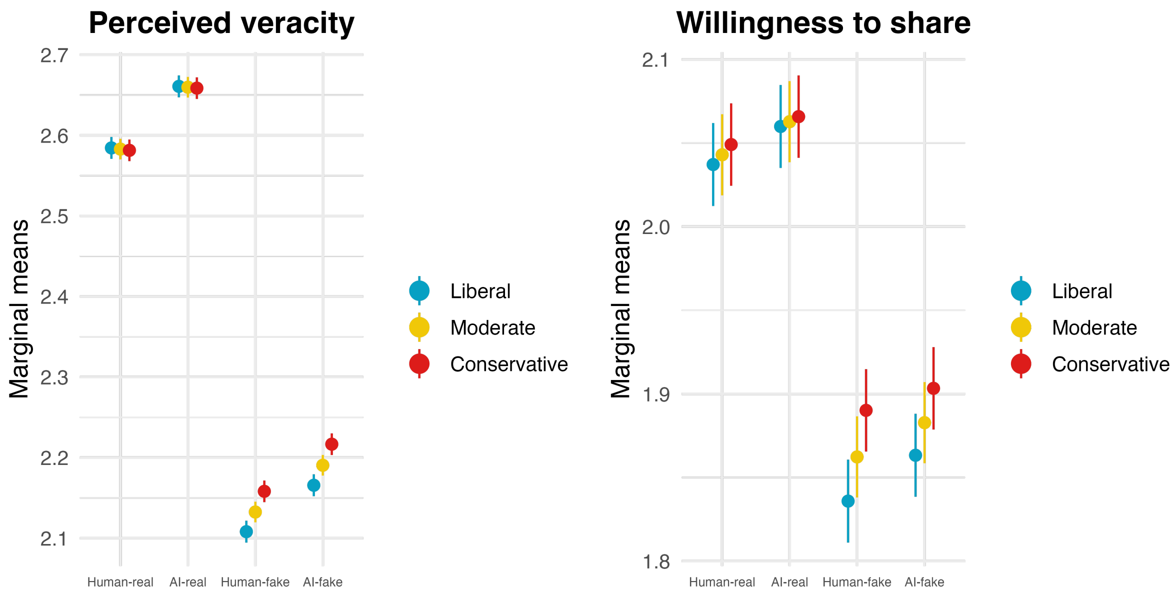

Fig S4. Marginal means of perceived veracity and willingness to share by ideological positions
Note: Higher values indicate greater perceived veracity and a higher willingness to share. Error bars represent standard errors. Differences between ideological positions were assessed using the mean and standard deviation.

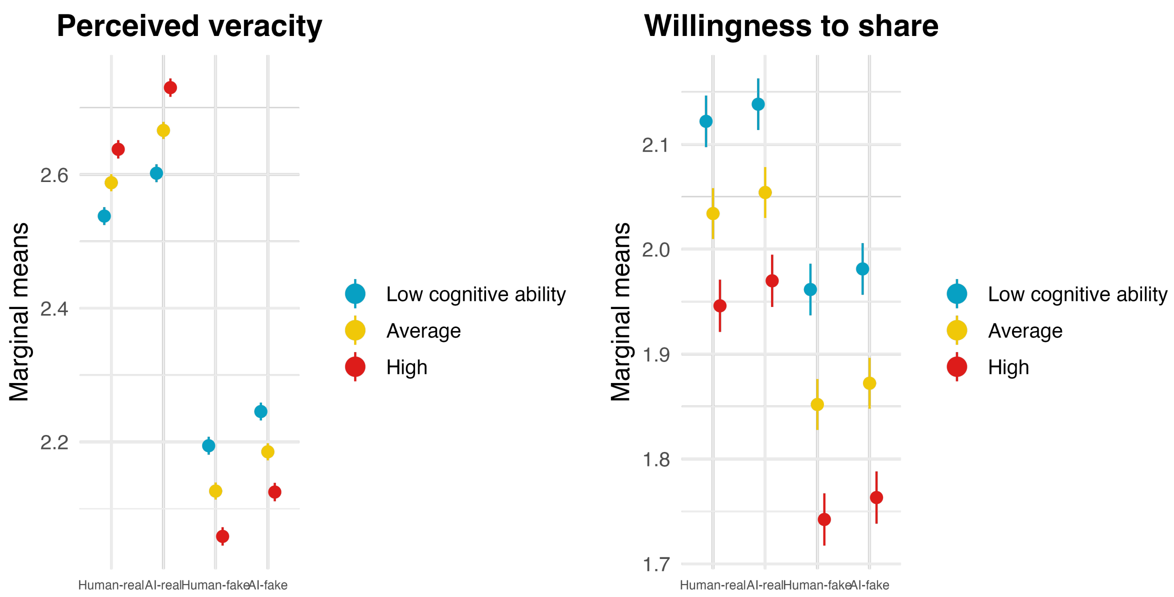

Fig S5. Marginal means of perceived veracity and willingness to share by cognitive ability levels
Note: Higher values indicate greater perceived veracity and a higher willingness to share. Error bars represent standard errors. Differences by cognitive ability were assessed using the mean and standard deviation.

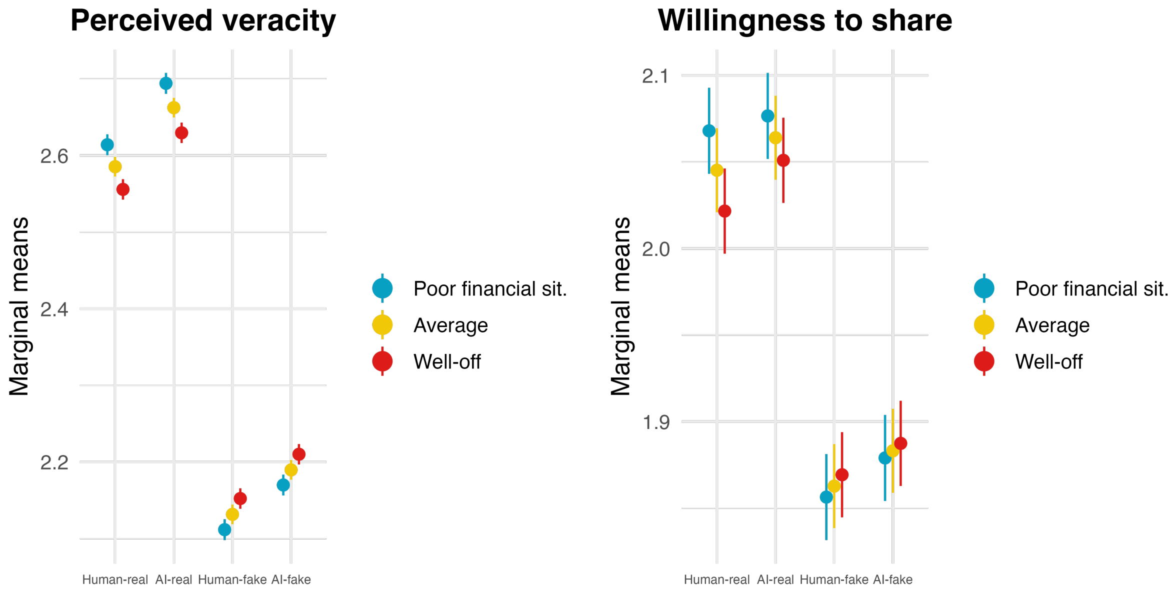

Fig S6. Marginal means of perceived veracity and willingness to share by financial situation
Note: Higher values indicate greater perceived veracity and a higher willingness to share. Error bars represent standard errors. Differences by financial situation were assessed using the mean and standard deviation.

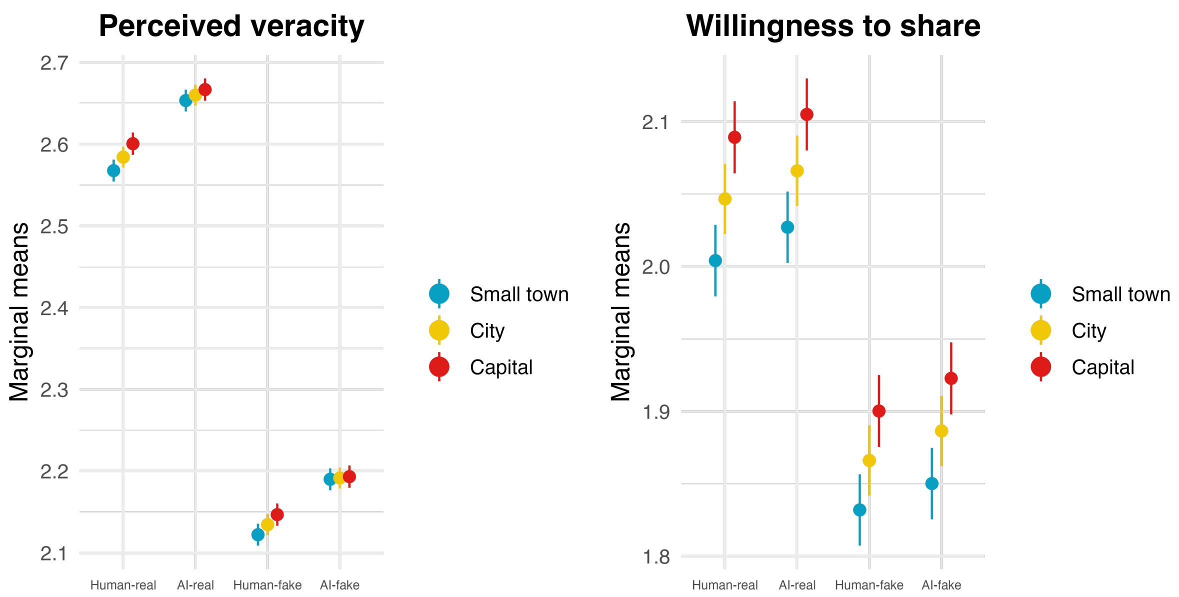

Fig S7. Marginal means of perceived veracity and willingness to share by settlement size
Note: Higher values indicate greater perceived veracity and a higher willingness to share. Error bars represent standard errors.

Country differences

Table S6. Results of the linear multilevel regression predicting perceived veracity in countries bordering Ukraine or Russia and in other European countries

	Non-bordering countries			Countries bordering Ukraine or Russia		
**Predictors**	**b (SE)**	**Std. Beta (SE)**	*p*	**b (SE)**	**Std. Beta (SE)**	*p*
Intercept	2.67 (0.02)	0.23 (0.02)	< 0.001	2.60 (0.03)	0.25 (0.02)	< 0.001
AI-real (ref.: Human-real)	0.07 (0.01)	0.08 (0.01)	< 0.001	0.08 (0.01)	0.09 (0.01)	< 0.001
Human-fake	-0.44 (0.01)	-0.49 (0.01)	< 0.001	-0.47 (0.01)	-0.52 (0.01)	< 0.001
AI-fake	-0.38 (0.01)	-0.42 (0.01)	< 0.001	-0.43 (0.01)	-0.47 (0.01)	< 0.001
Gender (ref.: Male)	-0.02 (0.01)	-0.03 (0.01)	0.001	-0.03 (0.01)	-0.03 (0.01)	0.011
Age	-0.30 (0.02)	-0.07 (0.00)	< 0.001	-0.15 (0.03)	-0.04 (0.01)	< 0.001
Secondary education (ref.: Low)	-0.04 (0.01)	-0.04 (0.01)	0.001	-0.02 (0.02)	-0.02 (0.02)	0.403
Tertiary education (ref.: Low)	-0.02 (0.01)	-0.02 (0.01)	0.093	-0.01 (0.02)	-0.02 (0.02)	0.534
Settlement size	0.04 (0.01)	0.01 (0.00)	< 0.001	-0.01 (0.02)	-0.00 (0.01)	0.680
Financial situation	-0.04 (0.01)	-0.01 (0.00)	0.001	0.04 (0.02)	0.01 (0.01)	0.037
Liberal-conservative	0.06 (0.02)	0.01 (0.00)	0.001	0.05 (0.03)	0.01 (0.01)	0.074
Cognitive ability	-0.01 (0.01)	-0.00 (0.00)	0.553	-0.02 (0.02)	-0.01 (0.01)	0.286
**Random Effects**						
σ^2		0.61			0.63	
τ_{00} (respid)		0.11			0.11	
τ_{00} (country)		0.00			0.00	
ICC		0.16			0.15	
N (respid)		18,805			6,800	
N (country)		20			7	
Observations		149,555			54,096	
R^2 (Marg. / Cond.)		0.071 / 0.219			0.079 / 0.218	

Table S7. Results of the linear multilevel regression predicting willingness to share in countries bordering Ukraine or Russia and in other European countries

	**Non-bordering countries**			**Countries bordering Ukraine or Russia**		
**Predictors**	**b (SE)**	**Std. Beta (SE)**	*p*	**b (SE)**	**Std. Beta (SE)**	*p*
Intercept	2.32 (0.03)	0.22 (0.03)	< 0.001	2.26 (0.07)	0.25 (0.07)	< 0.001
AI-real (ref.: Human-real)	0.02 (0.00)	0.02 (0.00)	< 0.001	0.02 (0.01)	0.03 (0.01)	0.002
Human-fake	-0.17 (0.00)	-0.19 (0.00)	< 0.001	-0.20 (0.01)	-0.23 (0.01)	< 0.001
AI-fake	-0.15 (0.00)	-0.17 (0.00)	< 0.001	-0.18 (0.01)	-0.20 (0.01)	< 0.001
Gender (ref.: Male)	-0.12 (0.01)	-0.14 (0.01)	< 0.001	-0.12 (0.02)	-0.13 (0.02)	< 0.001
Age	-0.88 (0.02)	-0.21 (0.01)	< 0.001	-0.47 (0.04)	-0.11 (0.01)	< 0.001
Secondary education (ref.: Low)	-0.12 (0.02)	-0.13 (0.02)	< 0.001	-0.08 (0.03)	-0.09 (0.04)	0.010
Tertiary education (ref.: Low)	-0.12 (0.02)	-0.14 (0.02)	< 0.001	-0.15 (0.03)	-0.16 (0.04)	< 0.001
Settlement size	0.16 (0.02)	0.06 (0.01)	< 0.001	0.00 (0.03)	0.00 (0.01)	0.967
Financial situation	-0.04 (0.02)	-0.01 (0.01)	0.033	-0.00 (0.03)	-0.00 (0.01)	0.936
Liberal-conservative	0.08 (0.03)	0.02 (0.01)	0.002	0.03 (0.04)	0.01 (0.01)	0.444
Cognitive ability	-0.31 (0.02)	-0.10 (0.01)	< 0.001	-0.35 (0.03)	-0.11 (0.01)	< 0.001
**Random Effects**						
σ^2		0.35			0.37	
τ_{00} (respid)		0.36			0.33	
τ_{00} (country)		0.01			0.02	
ICC		0.51			0.49	
N (respid)		18,805			6,800	
N (country)		20			7	
Observations		149,555			54,096	
R^2 (Marg. / Cond.)		0.071 / 0.542			0.047 / 0.510	

The news items

Human – real

Ukraine invasion: Russia restricts social media access

Russian regulator slows down access to Twitter (X) across country amidst spat with Facebook over fact-checking and labelling of Russian media posts.

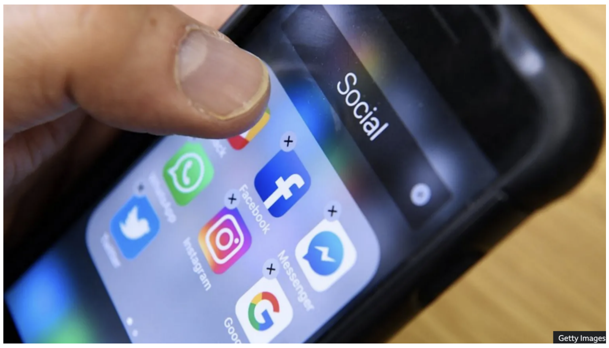

The U.S. Ukraine Mineral Deal

Volodymyr Zelensky says Ukraine is ready to sign a deal with the US over his country's mineral deposits.

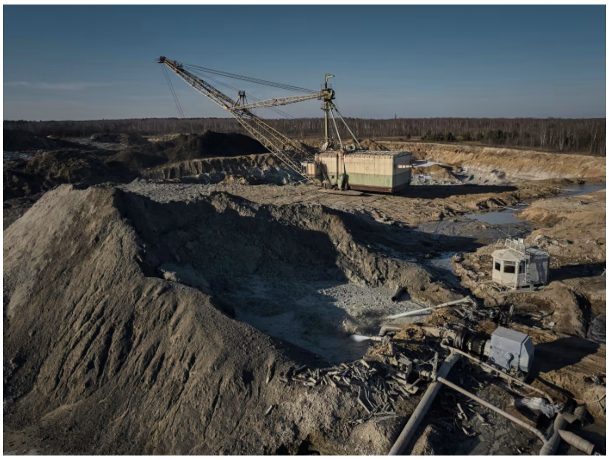

Zelensky opens door to same-sex civil partnerships in Ukraine

Ukrainian President Volodymyr Zelensky has opened the door to legalizing same-sex civil partnerships in the country, in response to a petition that called for equal marriage to be introduced in the war-ravaged nation.

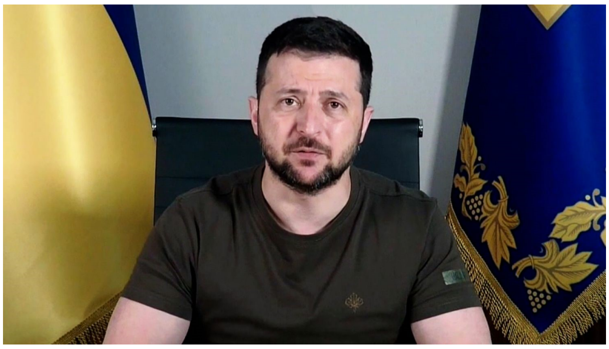

UK troops will not fight against Russia

Ukraine would instead be supported to "fight every street with every piece of equipment we can get to them" says Defence Secretary Ben Wallace.

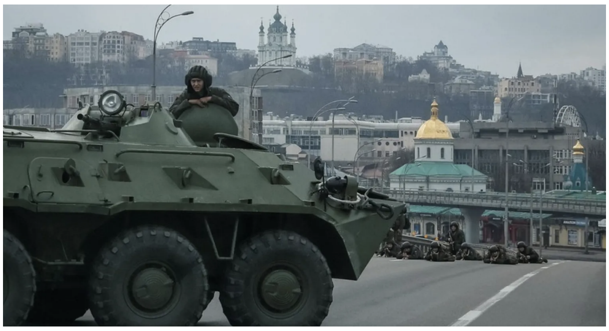

Germany announces new deal with Greece to send tanks to Ukraine

Germany will help get Soviet-era tanks from Greece to Ukraine by sending Athens modern German vehicles as a replacemen.

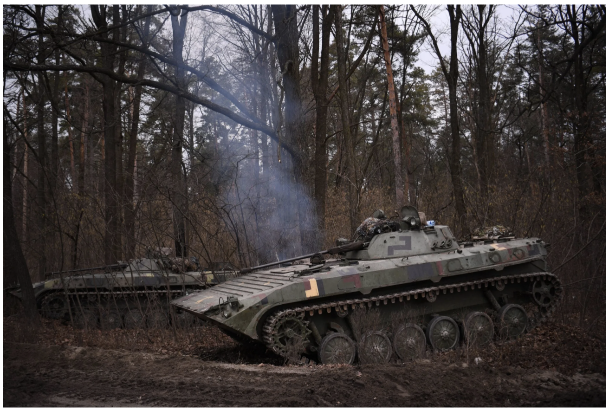

Zaporizhzhia: Ukraine suggests UN peacekeepers for nuclear plant

The inspectors recommended that a security zone be set up immediately to shield the facility, which is Europe's largest, from the fighting.

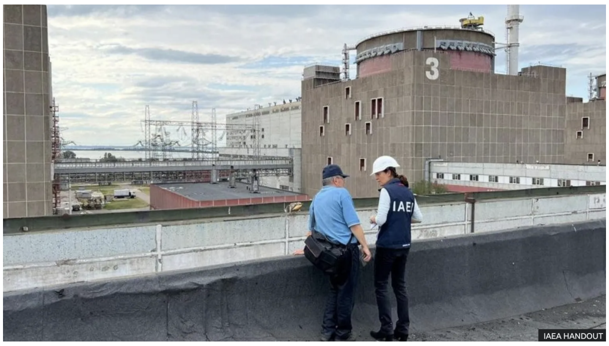

AI – real

Russia Restricts Access to Social Media Amid Ukraine Invasion

As the conflict with Ukraine escalates, Russian authorities limit platforms like Twitter, citing the spread of “false information” and threats to national stability.

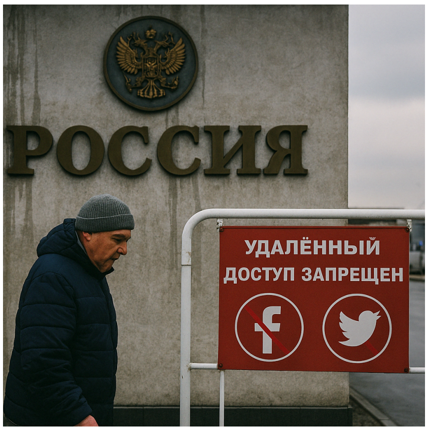

The U.S.–Ukraine Mineral Deal: Strategic Resources in Wartime Cooperation

A new agreement grants U.S. access to critical Ukrainian mineral reserves, strengthening bilateral ties and securing key materials for energy and defense industries.

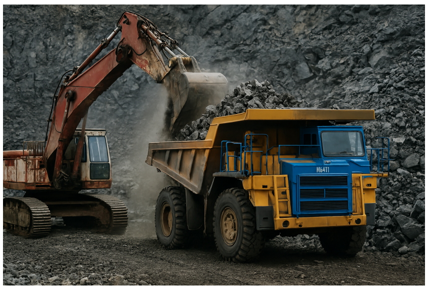

Zelensky Signals Support for Same-Sex Civil Partnerships in Ukraine

In a historic shift, Ukraine’s president expresses openness to legally recognizing same-sex unions, citing human rights and wartime unity.

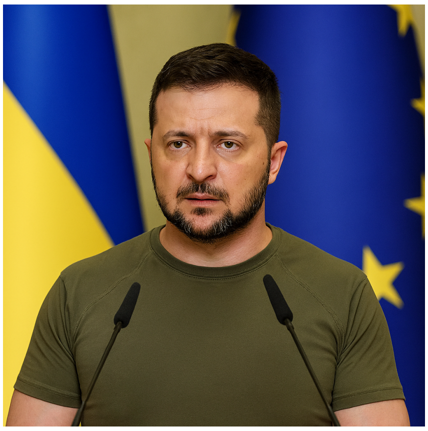

UK Confirms Troops Will Not Engage in Combat Against Russia

British officials reiterate that while support for Ukraine remains strong, there are no plans to deploy UK forces in direct conflict with Russian troops.

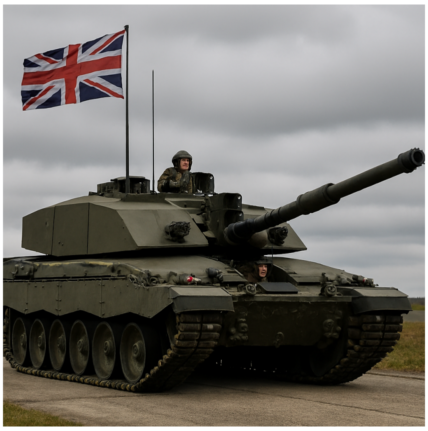

Germany and Greece Strike Tank Deal to Boost Ukraine's Defense

Under a new agreement, Germany will supply modern tanks to Greece, which in turn will transfer Soviet-era equipment to support Ukraine's ongoing war effort.

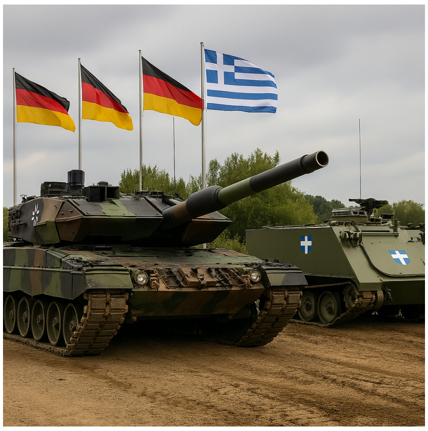

Ukraine Proposes UN Peacekeepers for Zaporizhzhia Nuclear Plant

Amid rising safety concerns at the Russian-occupied facility, Kyiv calls for a UN peacekeeping mission to protect the site and prevent a potential nuclear disaster.

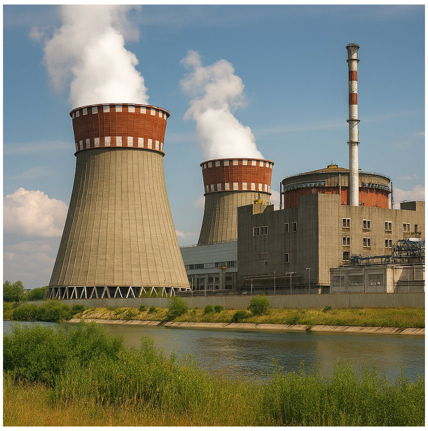

Human – fake

Ukraine banned Trump's Social media site

Ukrainian President Volodymyr Zelensky has reportedly blocked access to Truth Social, the social media platform launched by U.S. President Donald Trump.

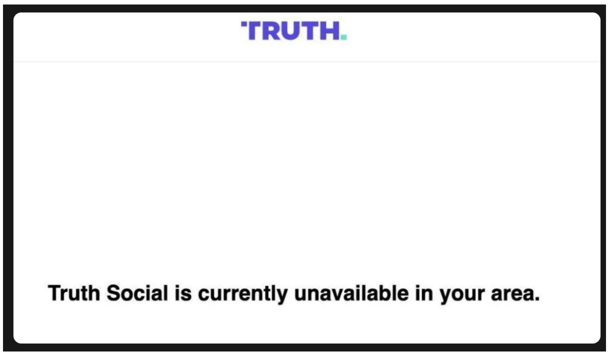

Russia Uncovers U.S. Military Bio-Weapons Labs in Ukraine

Russia claims it has found evidence of U.S.-funded labs in Ukraine developing dangerous pathogens and delivery systems, including infected mosquitoes and migratory birds.

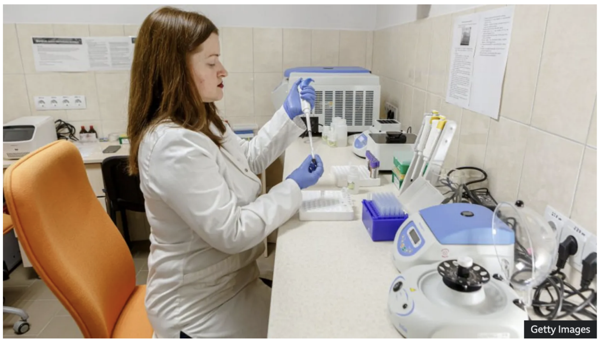

They officially added Ukraine to the pride flag

The blue and yellow of Ukraine's flag were incorporated into the iconic LGBTQ Pride design as a sign of solidarity.

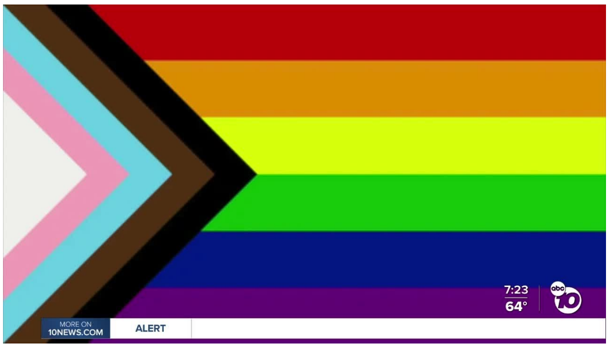

Mexican cartel obtained US weapons destined for Ukraine

Mexican TV reports that dozens of U.S. AT-4 weapons systems, which were originally shipped to Ukraine, have been purchased by Cartel Golfo in Mexico.

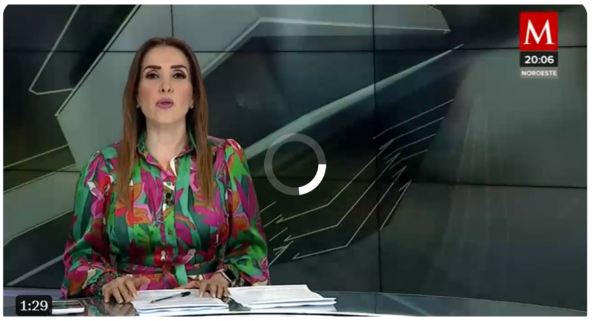

Greece committed more aid to Ukraine than France, Italy

Greece committed to give 260 million euros!! And for a measure of comparison: France committed to give 16 million (sixteen) and Italy 11 million!!!

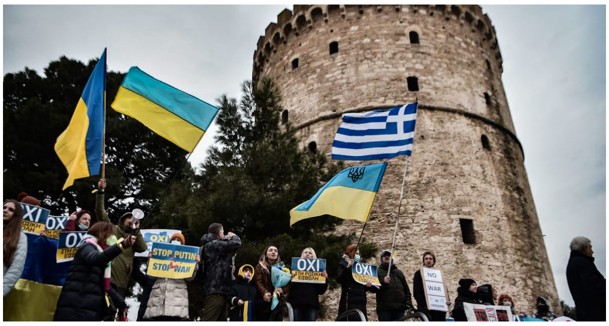

Przemyśl on Edge as Non-Ukrainian Migrants Arrive Amid War Refugee Wave

With the ongoing conflict in Ukraine driving mass displacement, the Polish border town faces new challenges as men from Africa and the Middle East seek entry alongside Ukrainian refugees, sparking local fears and debate.

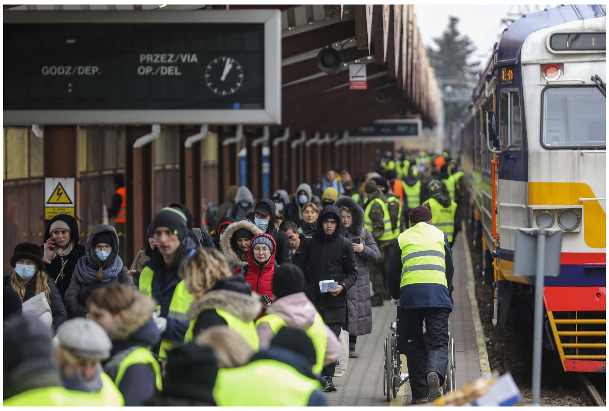

AI – fake

Ukraine Blocks Access to Trump's Truth Social

Authorities restrict the U.S. president's social media platform, citing national security considerations.

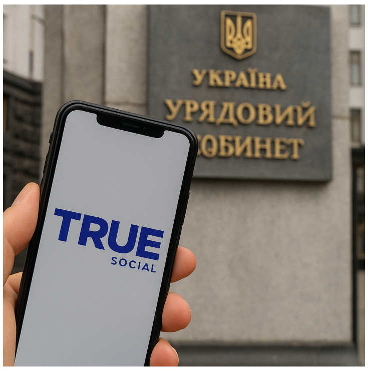

Russia Claims Discovery of U.S. Bio-Weapons Labs in Ukraine

Moscow alleges the presence of U.S.-funded biological research facilities operating on Ukrainian soil, escalating tensions over military-biological activity.

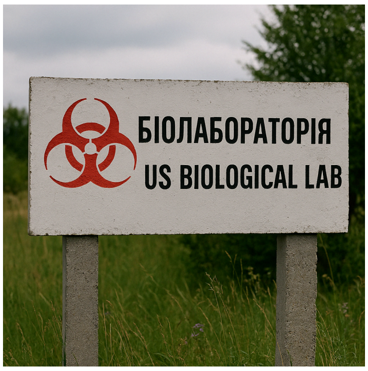

Ukraine's Colors Join the Pride Flag in a Bold Symbol of Solidarity

The blue and yellow of Ukraine's flag have been woven into the LGBTQ Pride flag, merging national identity with a global message of unity and resilience.

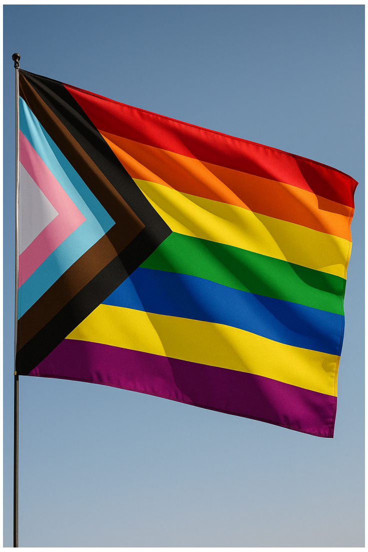

Mexican Cartel Acquires U.S. Weapons Originally Sent to Ukraine

Mexican media report that Cartel del Golfo has obtained U.S.-supplied AT-4 systems intended for Ukrainian forces, raising concerns over arms diversion.

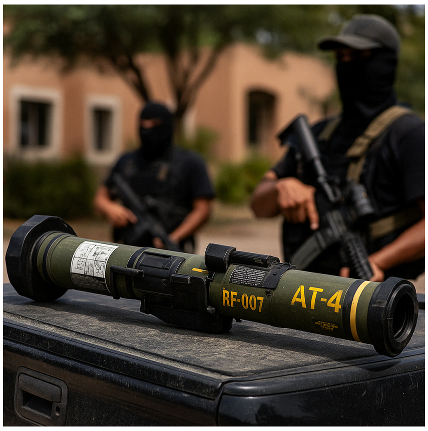

Greece Pledges More Aid to Ukraine Than France and Italy

Despite its smaller economy, Greece has outpaced major EU powers in military and humanitarian support to Ukraine.

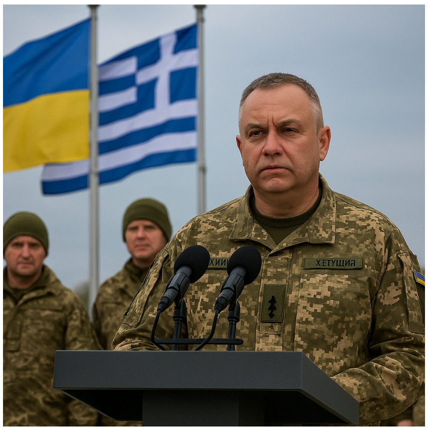

Border Tensions in Przemyśl Amid Influx of Migrants Claiming Ukrainian Refugee Status

As the Russo-Ukrainian war continues, residents of the Polish border town report growing unease over the arrival of undocumented men from Africa and the Middle East, raising concerns about security and strained resources.

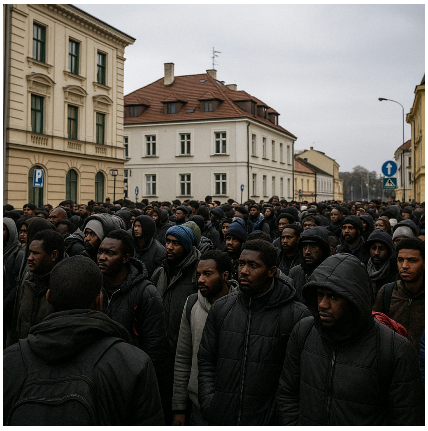

Question wordings

Q1. To what extent do you believe the news item you just read is real?

1. Does not seem real at all
2. Does not seem real
3. It seems real
4. It seems completely real

Q2. How likely is it that you would share the news item you have just read on social media?

1. Definitely would not
2. Probably would not
3. Probably would
4. Definitely would

Thank you for your responses. We would now like to ask your opinion on a different topic.

Sport is not merely a leisure activity, but a fundamental part of a healthy lifestyle. Physical activity supports good physical health and promotes mental well-being. Beyond physical benefits, sport also enhances social skills and teamwork, which are essential life competencies.

To confirm that you have carefully read all instructions, please select **surfing** as your answer to the next question.

Q3. What is your favourite sport?

1. Football
2. Basketball
3. Volleyball
4. Hockey
5. Surfing
6. Jogging
7. Other

Please note that some of the news items you have just read contained false claims and that we also included news items created by artificial intelligence in the experiment. We will now proceed with questions related to various public affairs.

Q4. Overall, how interested are you in politics?

1. Not interested at all
2. Not interested
3. Somewhat interested
4. Interested
5. Very interested

Q5. How would you describe your political orientation?

1. Strongly left-wing
2. Rather left-wing
3. Centrist
4. Rather right-wing
5. Strongly right-wing

Q6. How would you describe your ideological orientation?

1. Strongly liberal
2. Rather liberal
3. Centrist
4. Rather conservative
5. Strongly conservative

Cognitive Reflection Test

Q7. A bat and a ball cost \$1.10 in total. The bat costs \$1.00 more than the ball. How much does the ball cost? ________________ cents

Q8. If it takes 5 machines 5 minutes to make 5 widgets, how long would it take 100 machines to make 100 widgets? ________________ minutes

Q9. In a lake, there is a patch of lily pads. Every day, the patch doubles in size. If it takes 48 days for the patch to cover the entire lake, how long would it take for the patch to cover half of the lake? ________________ days

Sociodemographic Questions

Q10. What is your gender?

1. Male
2. Female
3. Other / Prefer not to say

Q11. What is your age? ________________ years

Q12. What is your highest level of education completed?

1. Primary
2. Secondary
3. Tertiary

Q13. What type of settlement do you live in?

1. Big city
2. Suburb of a big city
3. Town or small city

4. Village
5. Rural property or farm

Q14. How would you describe your household's current financial situation?

1. We are finding it very difficult to live on our current income
2. We are having some difficulties living on our current income
3. We are getting by on our current income
4. We live comfortably on our current income
